# Introduction of short course treatment for latent tuberculosis infection at a primary care facility for refugees in Winnipeg, Canada: A mixed methods evaluation

**DOI:** 10.3389/fpubh.2022.1064136

**Published:** 2023-01-16

**Authors:** Claudyne Chevrier, Mariana Herrera Diaz, Zulma Vanessa Rueda, Shivoan Balakumar, Margaret Haworth-Brockman, Diana Marcela Marin, Afsaneh Oliver, Pierre Plourde, Yoav Keynan

**Affiliations:** ^1^National Collaborating Centre for Infectious Diseases, Winnipeg, MB, Canada; ^2^Maestría en Epidemiología, Fundación Universitaria del Área Andina, Bogotá, Colombia; ^3^Department of Medical Microbiology and Infectious Diseases, University of Manitoba, Winnipeg, MB, Canada; ^4^Facultad de Medicina, Universidad Pontificia Bolivariana, Medellín, Colombia; ^5^Department of Community Health Sciences, University of Manitoba, Winnipeg, MB, Canada; ^6^BridgeCare Refugee Health Clinic, Winnipeg Regional Health Authority, Winnipeg, MB, Canada; ^7^Winnipeg Regional Health Authority, Winnipeg, MB, Canada; ^8^Department of Internal Medicine, University of Manitoba, Winnipeg, MB, Canada

**Keywords:** tuberculosis, tuberculosis infection, short term treatment, low incidence countries, refugee health

## Abstract

**Background:**

The World Health Organization (WHO) End TB strategy document ‘Toward tuberculosis elimination: an action framework for low incidence countries'—like Canada— identifies screening and treatment of latent tuberculosis infection (LTBI) for groups at increased risk for TB disease as a priority, including newcomers from endemic countries. In 2015, the clients-centered model offered at a primary care facility for refugees, BridgeCare Clinic, Winnipeg, Canada was evaluated. The model included LTBI screening, assessment, and treatment, and originally offered 9-months of isoniazid as treatment. This mixed methods evaluation investigates LTBI program outcomes since the introduction of two short-course treatment regimens: 4-months of rifampin, and 3-months of isoniazid and rifapentine.

**Methods:**

This study combined a retrospective analysis of program administrative data with structured interviews of clinic staff. We included LTBI treatment eligibility, the treatment regimen offered, treatment initiation, and completed treatment from January 1, 2015 to August 6, 2020.

**Results:**

Seven hundred and one people were screened, and infection rates varied from 34.1% in 2015 to 53.3% in 2020. Most people living with LTBI came from high TB burden countries in Africa and South-East Asia WHO regions and were younger than 45 years old. Treatment eligibility increased 9% (75% in 2015 to 86% in 2016–2020) and most people diagnosed with LTBI took the short course treatments offered. There was an increase of 14.5% in treatment initiation (75.6 vs. 90.1%), and an increase of 8% in treatment completion (82.4 vs. 90.4%) after short-course regimens were introduced. The final model showed that the treatment regimen tends to affect the frequency of treatment completion, but there are other factors that influence this outcome, in this population. With the new treatments, BridgeCare Clinic achieved the 90% of treatment coverage, and the 90% treatment completion rate targets recommended in the End TB Strategy. Qualitative interviews with clinic staff further affirm the higher acceptability of the new treatments.

**Conclusion:**

While these results are limited to government-sponsored refugees in Winnipeg, they highlight the acceptability and value of short-course LTBI treatment as a possibility for reaching End TB targets in primary care settings.

## 1. Introduction

Tuberculosis (TB) is one of the leading causes of mortality by an infectious agent worldwide, but in Canada it is primarily a disease of inequity and opportunity (1–3). As seen in other high-income countries, TB disease (TBD) burden in Canada's overall population is low (4.7 per 100,000 in 2020), while specific subpopulations experience disproportionately high rates (3, 4). Among these groups are refugees and newcomers who have recently migrated from countries with high TB incidence (1, 5, 6). In 2019, TB among foreign-born persons accounted for 74.2% of reported cases (incidence rate of 15.8 per 100,000 population) in Canada (4), with the absolute number of foreign-born persons diagnosed with TBD continuing to steadily increase, despite the incidence rate staying relatively stable since 2007 (1). In Manitoba's capital, Winnipeg, the foreign-born population accounted for 71.6% of TBD cases from 2013 to 2016 (7).

The World Health Organization (WHO) framework for low-incidence countries recommends eight areas of action for countries such as Canada, where the majority of people diagnosed with TBD result from the progression of latent tuberculosis infection (LTBI) rather than recent transmission, and a high proportion of cases result from cross-border migration (2).

In order to achieve the 2030 TBD elimination goals set by the United Nations (UN) and WHO (8), a concerted effort is needed to improve LTBI screening and treatment cascades for populations at increased risk of TB disease to prevent disease progression (2, 4, 9–12). Questions remain, however, regarding the best strategies to reduce LTBI burden in migrant and urban populations (13–17). In addition to general concerns about treatment toxicity and inconsistent treatment success rates with LTBI treatment (2), one significant concern is that the low incidence of TBD in Canada has resulted in a decline in general TBD management knowledge and an associated concentration of clinical expertise to a small number of physicians and nurses in the country (2, 18, 19). While some jurisdictions have responded to this by developing centralized TB programs with specialist clinics (20–23) and virtual services (24), other jurisdictions have relied on staff guidance, education and training to support decentralized TB management across primary health care systems (18, 25). The latter, while providing additional promise for holistic and integrated TB care (20, 23, 25), has shown uneven success in terms of TB program outcomes (13, 16, 26). It is therefore, important to understand the contexts and settings for care when looking at LTBI treatment outcomes.

Treatments for LTBI have traditionally relied on 6 to 9 months of isoniazid (9 INH), based on evidence for its clinical efficacy, and on the recommendation of the WHO (2, 12). However, hepatoxicity and low adherence have been long-time concerns. Since 2020, the WHO also recommends as first-line options 3-month regimen of weekly rifapentine plus isoniazid, or 3 months of daily isoniazid plus rifampicin, or 4 months of rifampicin alone over isoniazid monotherapy for tuberculosis preventive treatment in all TB burden settings (12, 27). These shorter, better tolerated treatments using rifamycin are proving safe and effective (28, 29).

This study builds on the results of a previous mixed methods evaluation of a promising LTBI treatment program at a primary care facility for refugees in Winnipeg, Canada – BridgeCare Clinic (25). The original study, conducted by Benjumea et al. in 2015–2016, reported the clinic's positive LTBI treatment outcomes (75% acceptance; 80% completion) and programmatic facilitators and barriers to treatment success. This study investigates treatment outcomes following the introduction of two short-course treatment regimens in 2017: 4 months of rifampin (4RMP) and 3 months of isoniazid and rifapentine (3 HP).

## 2. Materials and methods

To provide contextualized results that can inform LTBI program planning and delivery in Canada and other low-incidence countries in the world, we used a mixed methods evaluation study design to answer the following research questions: (1) Have short course LTBI treatment regimens (4 RMP and 3 HP) improved treatment outcomes compared with the previous regimen of 9 INH at BridgeCare Clinic?; (2) Did overall TBI program outcomes at BridgeCare Clinic improve after the introduction of short course treatment regimens 4 RMP and 3 HP?; and (3) What changes, challenges and considerations were involved with the introduction of 4 RMP and 3 HP regimens at BridgeCare Clinic?

### 2.1. Design

This study employed a concurrent embedded mixed methods research design (30) combining a retrospective analysis of program administration data with qualitative, structured interviews with clinic staff. Data were collected at the same time and combined during the analysis to provide a comprehensive understanding of treatment outcomes and the factors that contribute to those outcomes in the BridgeCare refugee population. The retrospective analysis of program administration data was used to answer research questions 1 and 2, and the interview data primarily answers question 3. However, the data from the interviews also provide context for the results seen in the administration data.

### 2.2. Setting

BridgeCare Clinic is a primary healthcare facility for government-assisted refugees in Winnipeg, Manitoba, Canada (31). According to BridgeCare clinic protocol, a person is eligible for LTBI screening with IGRA if they are between the ages of 18 and 49 years old and come from a country of birth or country of transition that is considered a TB endemic country (country with more than 30 cases of TB per 100,000 people every year) (32). Blood was drawn from participants at the laboratory located in BridgeCare clinic and transported to Cadham Provincial Laboratory in Winnipeg, Manitoba for QuantiFeron-Gold Plus (QTF-Plus) (QIAGEN Inc, Germantown, MD, USA) testing. The results were communicated back to the clinic for care management. In 2016, BridgeCare Clinic began offering 4 RMP/ 3 HP for clients eligible for LTBI treatment, replacing the long 9 INH treatment. However, some clients still received 9 INH treatment, although no reasons for long-course treatment were provided in the medical records we used.

BridgeCare Clinic is located in the capital city of the Prairie province of Manitoba, in Canada. The 2021 census put the population of Winnipeg just under 750 000 people (33). It also noted that the immigrant population (defined as “persons who are, or who have ever been, landed immigrants or permanent residents. Such persons have been granted the right to live in Canada permanently by immigration authorities”) was 201,040 people. The census defines “refugee” as “immigrants who were granted permanent resident status on the basis of a well-founded fear of returning to their home country” and reports 21,840 in the same period (33).

### 2.3. Quantitative data description

We used de-identified tuberculosis program data from Manitoba's Winnipeg Regional Health Authority from January 1, 2015, to August 6, 2020, for the quantitative analysis. Sociodemographic variables (sex, age, and country of birth, and years of attendance at BridgeCare Clinic) were used to describe the individuals with LTBI. The variables “WHO region” (34) and “WHO-TB burden” (35) were categorized according to the client's country of birth.

Only clients with IGRA test-positive results and a complete medical form were included in the analysis of treatment outcomes. We included LTBI treatment eligibility, the treatment regimen offered, treatment initiation, and completed treatment. The information related to the treatment received for each client is available only for those who initiated treatment.

### 2.4. Operational definitions

Definitions of TB treatment and outcomes were based on the WHO TB treatment guidelines 4^th^ edition (37), and the definitions used by the BridgeCare Clinic.

Treatment completion: A person diagnosed with TB who completed treatment without evidence of failure BUT with no record to show that sputum smear or culture results in the last month of treatment and on at least one previous occasion were negative, either because tests were not done or because results are unavailable. Treatment completion at BridgeCare clinic was defined as receiving ≥80% of doses within the time corresponding for each treatment regimen (25, 36).

Treatment failed: A person diagnosed with TB whose sputum smear or culture is positive at month 5 or later during treatment.

Lost to follow-up: A person diagnosed with TB who did not start treatment or whose treatment was interrupted for 2 consecutive months or more.

### 2.5. Quantitative analysis

All statistical analyses were performed using STATA v.14 (Stata Corp, College Station, TX, USA). Descriptive statistics (*n* [%]) were used to report the variables of interest. The prevalence and the 95% confidence intervals were used to report the IGRA results from the population screened at BridgeCare Clinic from 2015 to 2020. We evaluated the proportion of completion treatment among the years, and the treatments (short and long regimens).

To evaluate the association between the treatment (short or long regimens) and the treatment completion we used the Chi-squared test. We evaluated the interaction between sex and age on those associations using the Mantel-Haenszel test. In addition, to analyze whether the overall LTBI treatment outcomes at BridgeCare Clinic improved after the introduction of short course treatment regimens 4 RMP and 3 HP, we separated the clients in two groups. The first group (before) included clients attending BridgeCare Clinic during 2015 when only 9 INH was available (before). The second group (after) included clients attending the clinic from 2016 to 2020 when both regimens (short regimens and 9 INH) were available.

To evaluate the association between the cascade outcomes (eligibility, initiation, and completed treatment) and the groups (before and after) we used a Chi-squared test. We evaluated the interaction of sex and age on those associations using a Mantel-Haenszel test. We also reviewed and described the reasons reported by the BridgeCare medical team for the non-eligibility, non-acceptance, and non-completion of the LTBI treatment.

Subsequently, to estimate the effect of the treatment (short or long regimens) on the proportion of completed treatment (yes/no/unknown), adjusting for other variables, we used a multinomial regression model. To control for age, year of treatment, and cohort effect on the outcome, we included these variables in each model. We ran two models, considering the relationship between year of birth and birth cohort and the impossibility of using both in the same model because of their collinearity. In the first model we used the year of treatment initiation and the year of birth for each participant. In the second model we included the year of treatment initiation and the birth cohort of each person diagnosed with TB and an interaction term for these variables. In both models we also included the sex. We did not include the WHO region in the final models because this variable did not have an effect on the outcome.

Finally, we did sensitivity analyses to determine the robustness of the models, assuming all the unknown results in the categories of completed treatment as yes or no. We used the same variables included in the main model.

For all the analysis we considered statistical significance *p* < 0.05.

### 2.6. Qualitative data collection and analysis

A research assistant conducted four structured interviews in September and October of 2020 with BridgeCare clinical staff and program managers. The interviews were conducted over the phone with a standard script of questions. Initial contact with interview participants was made by email by the same research team member through a BridgeCare primary care nurse. Participants were chosen because of their role and knowledge of tuberculosis screening and treatment at BridgeCare Clinic.

Participants were informed of the research and went through an informed consent process and signed consent forms, as approved by the Health Research Ethics Board of the University of Manitoba. The interviewer recorded participants' comments by hand and participants were able to review the notes before their inclusion in the analysis. The interviews lasted an average of 45 min. A constant comparative analysis approach was used of the interview notes by the first author to help identify major themes and emergent categories. Emergent categories included description of program and participants, adherence, and acceptance of treatment, facilitating factors for continuation of treatment and overall perspectives on the TB treatment program. All documentation was anonymized and stored in locked files during the study and destroyed once the study was completed. Since the analysis was conducted on detailed notes from the interviews, the results presented below include very few direct quotations.

### 2.7. Ethics statements

The study received the approval of the Bannatyne Human Research Ethics Board at University of Manitoba, reference number: HS23661 (H2020:076). All records used in this research were provided by the clinic and excluded personal information that would allow for any identification of program clients.

## 3. Results

### 3.1. Retrospective analysis of program administrative data

Between January 2015 and August 2020, 701 of a total 2857 BridgeCare Clinic clients were screened for LTBI using IGRAs, and they were between 16 to 59 years old (a wider age range than the one mentioned as eligibility criteria). The frequencies of infection among those screened varied from 34.1% in 2015 to 53.3% in 2020 (Table 1). During those years, only seven clients had an indeterminate IGRA. The number of people screened at BridgeCare Clinic was lower in 2020 compared to preceding years due to fewer refugees entering Canada (and Manitoba) during the COVID-19 pandemic.

**Table 1:** Prevalence of IGRA results in population screened at BridgeCare Clinic, 2015–2020.

**IGRA results**	**2015**	**2016**	**2017**	**2018**	**2019**	**2020**
	***n*** = **176**	***n*** = **98**	***n*** = **85**	***n*** = **110**	***n*** = **202**	***n*** = **30**
Positive IGRA, [95% CI]	34.1 [27.4–42.3]	52.0 [42.2–61.8]	51.8 [41.2–62.2]	34.6 [26.1–43.8]	40.1 [33.5–47.0]	53.3 [35.6–70.5]
Negative IGRA, [95% CI]	65.3 [58.1–72.1]	46.9 [36.2–56.8]	47.1 [36.6–57.7]	63.6 [54.3–72.2]	59.4 [52.5–66.0]	43.3 [26.6–61.3]

IGRA, Interferon-Gamma Release Assays; 95% CI: 95% Confidence interval.

**Figure 1:**
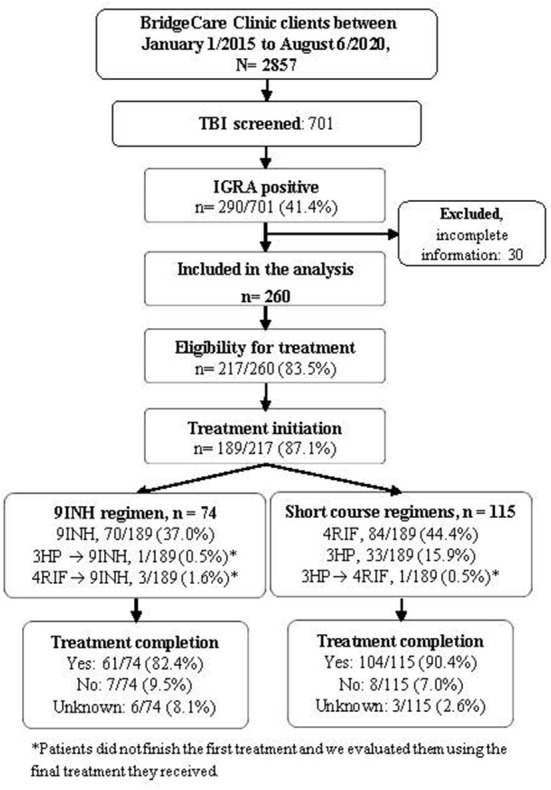
Tuberculosis infection cascade of care at BridgeCare Clinic. IGRA, Interferon-Gamma Release Assay; TBI: tuberculosis infection; 9INH, nine months of isoniazid; 4RIF, four months of rifampin; 3HP, three months of isoniazid (INH) and rifapentine (RPT); Final treatment regimen reflects the last medication taken and was used to evaluate treatment completion.

Thirty participants who received IGRA positive results but who had not been clinically evaluated at the time of this analysis due to the COVID-19 pandemic were excluded; data and results for 260 clients with positive IGRA were used in the subsequent analyses (Figure 1). Most people with LTBI who were screened at BridgeCare Clinic originated from high TB burden countries (>100 cases/100,000 inhabitants) in Africa and Eastern Mediterranean WHO regions, notably from Democratic Republic of Congo (96/260), Ethiopia (19/260), and Somalia (19/260). There were no clients with LTBI diagnosis from the Americas region. Most persons with a LTBI diagnosis were younger than 45 years old (mainly between 25 and 44 years old) (Table 2).

**Table 2:** Characteristics of individuals with tuberculosis infection diagnosis per year by sex, WHO region of origin, and age group.

	**2015 *n* = 60**	**2016 *n* = 51**	**2017 *n* = 44**	**2018 *n* = 29**	**2019 *n* = 60**	**2020 *n* = 16**
**LTBI by sex**	***n*** **(%)**	***n*** **(%)**	***n*** **(%)**	***n*** **(%)**	***n*** **(%)**	***n*** **(%)**
Female	28 (46.7)	30 (58.8)	19 (43.2)	12 (41.4)	31 (51.7)	8 (50.0)
Male	32 (53.3)	21 (41.2)	25 (56.8)	17 (58.6)	29 (48.3)	8 (50.0)
**LTBI by WHO region**						
Africa	47 (78.3)	45 (88.2)	36 (81.8)	22 (75.9)	46 (76.7)	13 (81.3)
Eastern Mediterranean	9 (15.0)	5 (9.8)	5 (11.46)	5 (17.2)	11 (18.3)	3 (18.8)
South-East Asia	4 (6.7)	1 (2.0)	2 (4.6)	0	1 (1.7)	0
Unknown	0	0	1 (2.3)	2 (6.9)	2 (3.3)	0
**LTBI by age, years**						
< 24	18 (30.0)	15 (29.4)	8 (18.28)	4 (13.8)	11 (18.3)	3 (18.8)
25–44	39 (65.0)	28 (54.9)	33 (75.0)	23 (79.3)	40 (66.7)	10 (62.5)
45–64	3 (5.0)	8 (15.7)	3 (6.8)	2 (6.9)	9 (15.0)	3 (18.8)

LTBI, latent tuberculosis infection; WHO, World Health Organization.

#### 3.1.1. Outcomes of short vs. long treatments

Among those with a positive result in the IGRA, 83.5% were eligible for preventive treatment, and 87.1% of them initiated the treatment (Figure 1). Overall, the frequencies of treatment completion by year were 79.4% (27/34) in 2015, 90.3% (28/31) in 2016, 91.4% (32/35) in 2017, 82.6% (19/23) in 2018, 92.3% (48/52) in 2019 and 78.6% (11/14) in 2020.

The frequency of people completing LTBI treatment with the short course regimens was higher (90.4%) compared to 9 INH regimen (82.4%), (*p*-value Chi-squared: 0.170) (Figure 1, Table 3). The association between the regimen and the frequency of treatment completion was affected by age, but not by sex. The characteristics of the people diagnosed with TB and the completion of treatment according to the treatment received (9 INH vs. short regimens) are reported in Table 3.

**Table 3:** Characteristics of the individuals who completed treatment, stratified by 9 INH and short course treatment regimens.

**Treatment completion**	**9 INH regime, *n* (%)** ***n* = 74**	**Short course regimes, *n* (%)** ***n* = 115**	***p*-value**
No	7 (9.5)	8 (6.9)	0.170^*^
Unknown	6 (8.1)	3 (2.6)	
Yes	61 (82.4)	104 (90.4)	
Sex	Treatment completed/*n*	Treatment completed/*n*	0.077^+^
Male	32/37 (86.49)	57/65 (87.7)	
Female	29/37 (78.4)	47/50 (94.0)	
Age, years	Treatment completed/*n*	Treatment completed/*n*	**0.038** ^+^
< 24	16/16 (100)	21/24 (87.5)	
25–44	39/51 (76.5)	74/81 (91.4)	
45–64	6/7 (85.7)	9/10 (90.0)	

LTBI, tuberculosis infection; 9 INH: 9 months of isoniazid;

+ *p*-value represents Mantel-Haenszel test;

* *p*-value represents Chi-squared tests.

IGRA: Interferon-Gamma Release Assay; LTBI: latent tuberculosis infection; 9 INH: 9 months of isoniazid; 4RIF: 4 months of rifampin; 3 HP: 3 months of isoniazid (INH) and rifapentine (RPT); Final treatment regimen reflects the last medication taken and was used to evaluate treatment completion.

When looking at the reasons why people diagnosed with TB did not complete different treatment regimens for LTBI, no reasons were given in 71.4% (5/7) of abandoned 9 INH treatments, and 75% (6/8) of incompletion were attributed to side effects for short course regimens. Only one person did not complete treatment due to transfer of care. The side effects reported in the medical form, for both treatments, included liver disturbance, mental health symptoms, nausea/vomiting, rash, and extreme fatigue.

#### 3.1.2. Overall LTBI program outcomes before and after the introduction of short-course treatment regimens

We analyzed the entire cascade of care, among the total people with LTBI who were screened at BridgeCare Clinic before (2015) and after (2016–2020) the introduction of short-course treatment regimens. From 2016 to 2020, the use of 9 INH decreased with 25.8% (40/155) of clients receiving 9 INH during those years. 54.2% (85/155) of the clients received 4 RIF regimen and 19.4% (30/155) received 3HP. Five clients changed their regimen received during the years analyzed.

**Figure 2:**
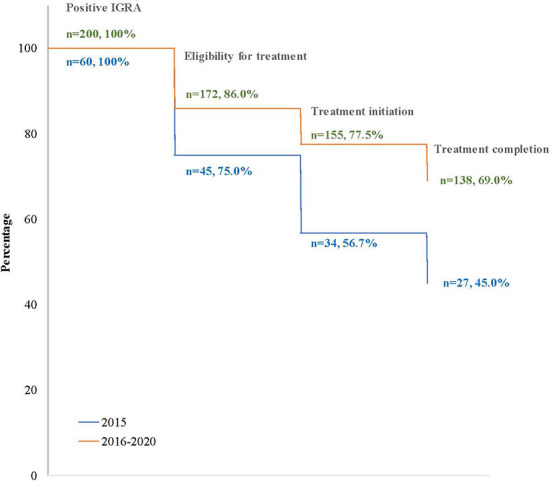
Losses (percentage) in the cascade of tuberculosis infection care in clients at BridgeCare Clinic with positive IGRA, before (2015) and after (2016–2020) introduction of the offer of short-course regimens.

Forty-five percent (27/60) of clients with LTBI completed treatment when only the long-course option was available (2015), and 60% (138/230) did after short-course regimens were introduced in 2016 (Figure 2).

Overall, after the introduction of short-course regimens there was an improvement in the LTBI program outcomes at BridgeCare Clinic. There was a higher proportion of clients eligible for treatment who initiated and completed treatment. In particular, 14.5% more clients initiated treatment among those eligible for treatment when short-course regimens were available (90.1 vs. 75.6%; *p*-value: 0.009), (Table 4).

**Table 4:** Characteristics of the individuals analyzed in the LTBI diagnosis cascade of care, before and after short course treatment introduction at BridgeCare Clinic.

**Outcomes of the cascade of care and characteristics of the individuals**	**Before, *n* (%)**	**After, *n* (%)**	***p-*value**
**Eligibility for treatment**	***n*** **=** **60**	***n*** **=** **200**	0.124^*^
Not candidate	10 (16.7)	20 (10.0)	
Unknown	5 (8.3)	8 (4.0)	
Eligible	45 (75.0)	172 (86.0)	
**Sex**			0.630^**+**^
Male	26/32 (81.2)	93/100 (93.0)	
Female	19/28 (67.9)	79/100 (79.9)	
**Age, years**			0.891^**+**^
< 24	14/18 (77.8)	35/41 (85.4)	
25–44	29/39 (74.4)	117/134 (87.3)	
45–64	2/3 (66.7)	20/25 (80.0)	
**Treatment initiation**	***n*** **=** **45**	***n*** **=** **172**	**0.009** ^*^
No	11 (24.4)	17 (9.9)	
Yes	34 (75.6)	155 (90.1)	
Sex			**0.032** ^**+**^
Male	22/26 (84.6)	80/93 (86.0)	
Female	12/19 (63.2)	75/79 (94.9)	
Age, years			0.098^**+**^
< 24	9/14 (64.3)	31/35 (88.6)	
25–44	25/29 (86.2)	107/117 (91.4)	
45–64	0/2	17/20 (85.0)	
**Treatment completed**	***n*** **=** **34**	***n*** **=** **155**	0.287^*^
No	4 (11.8)	11 (7.1)	
Unknown	3 (8.8)	6 (3.9)	
Yes	27 (79.4)	138 (89.0)	
Sex			0.511^**+**^
Male	19/22 (86.4)	70/80 (87.5)	
Female	8/12 (66.7)	68/75 (90.7)	
Age, years			**0.010** ^**+**^
< 24	9/9 (100)	28/31 (90.3)	
25–44	18/25 (72.0)	95/107 (88.8)	
45–64	0	15/17 (88.2)	

+ *p*-value represents Mantel-Haenszel test.

* *p*-value represents Chi-squared test.

The bold values indicate the overall values for the section.

Table 4 shows that there were differences in the proportion of treatment initiation, before and after the introduction of the short course regimens, in women compared to men (*p*-value _M−H_: 0.032**)**. There were also differences in the proportion of treatment completed, before and after, in the categories of age (*p*-value _M−H_: 0.010), (Table 4).

The reasons why individuals did not initiate treatment before and after the short-course introduction were mostly unknown in both groups. Known reasons for not initiating included dislike for taking medication and side effects. After the introduction of short course regimens, the main reason for treatment ineligibility was related to perceived fears around being or trying to get pregnant, and breastfeeding in the 2016–2020 cohort of women (Supplementary Table S1).

In addition, after evaluating the multinomial regression models, we found that the short treatment regimen, and female sex tend to positively affect the treatment completion proportion, but there are other factors affecting this association that were not evaluated in this analysis (Table 5). The cohort of birth and the year of birth of the clients do not show conclusive results in the models, with contrary effects (Supplementary Table S2). We got similar results when we did the sensitivity analyses, assuming the unknown category as completed or not completed treatment (Supplementary Tables S3, S4).

**Table 5:** Multinomial regression to evaluate the effect of the regimen of treatment, birth cohort, sex, and year of treatment on the frequency of treatment completion.

**Treatment complete**		**RRR**	**aRRR**	***p*-value**	**95% CI**
No (Ref outcome)		1	1	-	-
Yes	Short regimen	1.49	2.06	0.285	0.55–7.77
	Sex (Female)	0.97	1.12	0.846	0.36–3.40
	Year of treatment	0.97	0.96	0.928	0.46–2.03
	Cohort of birth	0.98	1.20	0.795	0.29–4.96
Unknown	Short regimen	0.43	0.62	0.673	0.07–5.67
	Sex (Female)	0.91	0.85	0.853	0.15–4.89
	Year of treatment	0.73	0.58	0.383	0.17–1.96
	Cohort of birth	0.89	0.54	0.552	0.07–4.10

The model includes an interaction term between variables year of the treatment initiation and cohort of birth, (*n* = 189). The first birth cohort includes those who were born up to and including 1960 and in 10-year periods thereafter. RRR: relative risk ratio; aRRR: adjusted relative risk ratio CI: confidence interval.

### 3.2. Results from structured interviews of clinic staff

#### 3.2.1. BridgeCare clients and families

The clinical and administrative staff at BridgeCare clinic who were interviewed reported few observed differences in the characteristics of clients screened for LTBI before and after the introduction of both short-term treatment options, including by countries of origin, age, family structure, and health concerns. They reported that the LTBI program usually sees younger families and a few older people. Family structure was noted to be consistent with some exceptions, such as a period of time when many Yazidi refugees, who were predominantly women without their husbands, were clients at the clinic in 2017. Overall, as one interviewee noted, “sometimes we see single mother families, sometimes large families.” One interviewee also made clear that with the advent of the COVID-19 pandemic, there was an abrupt stop in client intake because refugees, like everyone else, were no longer allowed to enter Canada, per public health orders.

#### 3.2.2. Acceptance, adherence, completion, and outcomes of treatment

No changes, or a slight increase, in acceptance and completion of treatment were noted by interviewees since the introduction of the new treatment options. BridgeCare staff viewed the introduction of the new treatments as having improved the receptivity to treatment overall because of their reduced duration, and one clinical staff mentioned noticing fewer missed appointments. The fewer side effects associated with the specific medications in the new treatments were identified as a factor influencing their acceptance, as well as their suitability for clients with liver inflammation from chronic hepatitis. The new treatments were also described as improving the ability to complete treatment within the 12-month period that clients are followed at BridgeCare.

In terms of challenges, it was noted that the new treatment medications can sometimes have more interactions with other medication, such as hormonal birth control and anti-hypertensives. It was also reported that there were no changes in effectiveness, with two clinical staff saying that they had not seen any person diagnosed with TB progress to TB disease.

No differences in adherence were reported by any staff between clients receiving 3HP through directly observed treatment (DOT) vs. self-administered treatment with 4-month rifampin. One participant stated that 3 HP DOT given weekly is more work for nurses, and another shared that they believe that it has contributed to improved continuity of care.

#### 3.2.3. Other factors facilitating the LTBI program

On-site lab sampling at BridgeCare Clinic was already noted in 2015 to be a facilitator for the program by making it possible to incorporate LTBI screening with all other screening (25). Staff members brought this up during interviews in this study, with one of them noting that given the language and cultural barriers facing certain clients, having to navigate the city and healthcare system to get certain tests— including IGRA— was sometimes extremely challenging. Additionally, one person noted that IGRA can be a difficult test to perform and having a laboratory staff on-site has likely reduced the number of cancelled tests due to error. On-site lab work has also made ongoing blood monitoring easier.

A second programmatic change at the clinic that one person noted, was that the electronic medical records had been updated to include more LTBI fields, which improved the ability of staff to trace client files.

#### 3.2.4. Overall opinion of program and the introduction of short-course treatments

The BridgeCare clinic staff reported that, from their perspective, the program is effective and has improved with the introduction of short course treatments. Among the positive points that contribute to this improvement, interviewees identified that the trust, comfort, and familiarity in the relationship between client and clinical staff improves the acceptance of treatment and also facilitates navigating the healthcare system for newcomers, for whom it can otherwise be very challenging. They also noted that the easy access to interpreters greatly facilitates care for people diagnosed with TB. As was seen before, two interviewees also highlighted that the fact that most client come from TB endemic countries, have more knowledge of TB, and are likely to have known people who had TB disease, may contribute to their high treatment acceptance rate.

Some barriers to the overall success of the program were also identified. One was the difficulty to evaluate the actual risk of TB disease in client without knowing how long they have had LTBI. This sometimes led to offering treatment as a default to avoid the possibility of TB reactivation and transmission in the near future. It was also mentioned that for BridgeCare's younger population, being pregnant or trying to become pregnant can prevent some women from accepting the treatment.

## 4. Discussion

This research project aimed to provide contextualized results regarding administration and completion of short-term treatments, to inform LTBI program planning and delivery in Canada and other low-incidence countries. With the introduction of short-term LTBI treatments, BridgeCare Clinic achieved the 90% of treatment coverage and the 90% of treatment completion rate (Table 3) targets identified by the End TB targets. We found no difference in LTBI screening and prevalence between males and females, nor by age, before and after the treatment changes.

Latent TB infection identification and treatment has been recognized as one of the most important points to achieve the TB elimination goal. The current guidelines recommend ryfamicin-based treatment regimens over longer-course treatment for LTBI (29). Our study reports the experience of a primary care clinic with a client-centered TB program that achieved two milestones after the introduction of short-term regimens for LTBI treatment: more than 90% of people with LTBI diagnosis and eligible for treatment started LTBI therapy. Clients receiving the short regimens completed treatment in >90% of cases, and in general terms, after introducing short regimens, this goal was almost achieved with 89% of clients completing treatment. Several papers have reported that short-term treatments have higher rates of completion (38–42). The interviews with BridgeCare Clinic staff also support this finding. Indeed, interviewees reported that the new treatment options, in their views, had increased the acceptability of treatment for their clients, noting that there were fewer missed appointments. They identified the reduced treatment duration, fewer side effects, and suitability for clients with chronic liver conditions as factors influencing acceptability of the treatment. It should be noted that, as indicated in Table 5, there were some side-effects with the shorter treatments. One of the main fears with isoniazid-based regimens is the hepatotoxicity (29, 43). This is alleviated by recent findings that show, for example, that 4 months of rifampin has lower risk of severe hepatotoxicity, lower costs and higher treatment completion, compared to 9 months of 337 isoniazid (44). Shorter regimens are being evaluated for TB preventative treatments, such as a 1-month regimen of daily isoniazid and rifapentine in certain key populations (45), or a 6-weeks 339 regimen of daily rifapentine (46).

Pillar 1 of the End TB Strategy advocates for integrated, client-centred care and prevention (8). The evaluation of the LTBI program at BridgeCare Clinic undertaken in 2016/2017 found that treatment can be delivered effectively in the primary care setting using an integrated, client-centered approach (25). The improvement in the treatment initiation and completion affirms previous findings showing the strength of the integrated LTBI care model offered at BridgeCare Clinic (25) and the potential of the improvement of LTBI screening and treatment cascades to prevent disease progression (2, 9, 12). As documented elsewhere (25), the trust, comfort and familiarity in the existing relationships with clinic staff were identified as contributing to the enhanced treatment uptake and completion.

Despite these milestones, there is a critical step within the cascade of care to improve: the number of people who start LTBI treatment among those with LTBI diagnosis. When we evaluated the total cascade of care, 67.39% of people with TB with LTBI initiated LTBI treatment. There are clients-specific factors associated to non-initiation of LTBI treatment and lack of adherence to LTBI treatment that require individualized interventions targeted to specific populations (47). It would be important to understand barriers and facilitators for refugees from starting LTBI treatment (17). Also, there are some strategies that can be considered by local health authorities that can reduce losses in the LTBI cascade, such as incentives, home visits, digital solutions, and clients reminders (48).

One critical factor that is not related to people living with TB, is the importance of continuing research and the development of new and shorter LTBI treatments with minimal side effects to increase the desirability and uptake of LTBI treatment, as evidenced by concerns or fears of side effects or the duration of the treatment being reported as the main reasons to not initiate treatment documented in our study.

There were no observed differences in LTBI screening, prevalence, or treatment completion between males and females before and after the treatment changes, but sex could affect the relationship between treatment completion and the availability of short regimens at BridgeCare clinic. However, after analyzing the multinomial model, this difference was not conclusive, and in some cases could do not exist as reported in previously published research done in the general population in Norway in 2016 (48). The majority of women who refused the treatment did so because of they were pregnant or planning to be and thus could not or were unwilling to take the medication. The information collected in this research project does not clarify if clients were followed up later to be offered the treatment again. Since BridgeCare Clinic only offers services to its clients for 1 year (25), follow-up beyond this timeframe falls outside the scope of their mandate.

The lack of difference between sexes before and after the introduction of the treatment changes can suggest a consistency in the approach by the BridgeCare staff and that there may be no family constraints on women getting care. Our clinical data and the data from the interviews do not allow us to comment on the potential gendered differences in the ability to get to the clinic, to manage drugs, to pay for drugs, or to negotiate family planning while on the treatment.

Our findings show a small decrease in the proportion of treatment completion in 2018 compared with the previous year. A possible explanation for this is the change in refugee demographics characteristics among the years, as reported by clinical staff. A systematic review reports that sociodemographic and cultural factors might influence the decision of migrant persons diagnosed with TB to initiate and complete treatment. Those factors include region of origin, employment status, difficulties in effectively communicating, stigma (17). The timeframe for this study may also reflects the effect of the COVID-19 pandemic on the TB program at BridgeCare clinic. A reduction in treatment completion frequency from 92.3% in 2019 to 78.6% in 2020 could be related to the COVID-19-related disruptions of health services, mobility restrictions, and lockdown measures in 2020 (49, 50). In fact, Bridgecare Clinic stopped doing IGRAs and did not offer LTBI therapy after March 2020. These disruptions have been reported worldwide, with the WHO documenting that they have affected over 84 countries, with 1.4 million fewer people estimated to have received TB care in 2020 (51). We advocate for the importance of evaluating whether the frequency of treatment completion continues to be similar to the values seen in 2020 and, for implementing new strategies to revert the impact of COVID-19 on the TB program at BridgeCare clinic and similar programs around the world. These external factors may have influenced the findings and affected the statistical significance reported in this study. We would argue that, much like the COVID-19 pandemic has encouraged public health policy makers and practitioners to focus on testing approaches that take into account the populational health context and prioritize population testing effectiveness over test sensitivity (52), it is important to consider the public health relevance of the findings presented in this paper.

### 4.1. Limitations

Our results come from a database generated by the clinical staff of BridgeCare Clinic, where several people were responsible for entering data and maintaining the registry during the years of interest of the study (49). This led to some information about treatment acceptance being written in greater or less detail. In addition, during these years, two information collection and storage systems were used; in 2018 the clinic migrated to a new collection system that contains more complete and detailed information on each clients. These two factors may have contributed to the quality of the data used for the analysis, and the latter contributed to the exclusion of certain clients from the analysis.

We considered that clients' background, education and other factors could differ between the two cohorts in the analysis. However, we have limited information about those variables from the medical records kept by BridgeCare Clinic. In the same way, the clinic does not have information about the potential regimens offered for each client before the date of treatment initiation.

The qualitative interviews were conducted with clinical and administrative staff of the clinic exclusively. This means that the perspectives of clients eligible for treatment and their experience regarding barriers and facilitators to treatment were not included. This is an opportunity for future research.

Our results only reflect the adult clients seen at BridgeCare clinic and cannot be generalized to all refugees in Manitoba.

Future studies should document the percentage of people with incipient tuberculosis, latent tuberculosis infection, and subclinical tuberculosis (50, 51), as well as the number of people who progress to tuberculosis diseases among those with tuberculosis infection.

## 5. Conclusion

Achieving 90% treatment coverage, and 90% treatment completion rate targets recommended in the End TB Strategy in a refugee population is notable and highlights the possibility of reaching the End TB targets in a primary care setting. While these results are limited to government-sponsored adult refugees in Winnipeg, Canada, they highlight the possibility of reaching End TB targets in a primary care setting and to successfully maintain a cascade of care. Of course, this does not provide us with an overall picture for all priority populations (such as Indigenous peoples). The results from this study indicate that short-course treatment regimens such as 4-months of rifampin (4 RMP), and 3-months of isoniazid and rifapentine (3 HP) can improve LTBI screening and treatment cascades in higher risk populations.

## Data availability statement

The data that support the findings of this study were provided by the Winnipeg Regional Health Authority but restrictions apply to the availability of these data, which were used under license for the current study, and so are not publicly available. Data requests can be made via the authors, who will seek appropriate permissions from the Winnipeg Regional Health Authority as needed.

## Ethics statement

The studies involving human participants were reviewed and approved by Bannatyne Human Research Ethical Board at University of Manitoba, reference number: HS23661 (H2020:076). The clients/participants provided their written informed consent to participate in this study.

## Author contributions

Substantial contributions to the conception or design of the work: SB, MD, YK, and MH-B. Acquisition of data: SB. Analysis and interpretation of data: MD, SB, ZR, CC, and DM. Drafting the article and revising it critically for important intellectual content: CC, MD, YK, SB, MH-B, ZR, PP, and DM. Final approval of the version to be published and agreement to be accountable for all aspects of the work in ensuring that questions related to the accuracy or integrity of any part of the work are appropriately investigated and resolved: CC, MD, YK, MH-B, SB, ZR, PP, and DM. All authors contributed to the article and approved the submitted version.
