# Supplementary material for: Introduction of short course treatment for latent tuberculosis infection at a primary care facility for refugees in Winnipeg, Canada: A mixed methods evaluation
Source: Front Public Health. 2023 Jan 16;10:1064136. doi: 10.3389/fpubh.2022.1064136 (PMC9885188; doi:10.3389/fpubh.2022.1064136)
Supplement: Supplementary file 1 [file Data_Sheet_1.docx]

**Supplementary material.**

**Table S1**. Reasons for not being eligible and not starting tuberculosis infection treatment, before and after the short course regimens were available in 2016. From 2016 to 2020 some clients received 9INH treatment

|  | **Before (2015), n** | **After (2016-2020), n** |
| --- | --- | --- |
| Reason for being not eligible for LTBI treatment, n=30 | 10 | 20 |
| Breastfeeding | 0 | 2 |
| Active tuberculosis | 2 | 1 |
| Medical issue | 3 | 4 |
| Pregnancy | 4 | 9 |
| Treatment on hold for COVID-19 pandemic | 0 | 1 |
| Trying to get pregnant | 0 | 1 |
| Unspecified | 1 | 2 |
| Unknown eligibility, n =13 | 5 | 8 |
| Changed health care provider | 0 | 3 |
| Lost to care | 0 | 3 |
| Moved | 5 | 2 |
| Reason to not start LTBI treatment, n= 28 | 11 | 17 |
| Declined - does not like taking medication | 0 | 2 |
| Declined - family advised against | 0 | 1 |
| Declined - length of treatment | 0 | 1 |
| Declined - side effects | 0 | 2 |
| Declined - travel | 0 | 1 |
| Declined - unspecified | 11 | 10 |
| Reason to not complete LTBI treatment, n=15 | 4 | 11 |
| Transfer of care | 0 | 1 |
| Liver disturbance - ALT increased | 0 | 2 |
| Other side effects - mental health symptoms. | 0 | 2 |
| Other side effects - nausea, vomiting | 0 | 1 |
| Other side effects - rash | 0 | 1 |
| Side effects - extreme fatigue | 0 | 1 |
| Side effects - unspecified | 0 | 1 |
| Unknown | 4 | 2 |

**Table S2**. Multinomial regression to evaluate the effect of the regimen of treatment, year of birth, sex, and year of treatment on the frequency of treatment completion.

| **Treatment complete** |  | **RRR** | **aRRR** | **p value** | **95% CI** |
| --- | --- | --- | --- | --- | --- |
| No (Ref outcome) |  | 1 | 1 | - | - |
| Yes | Short regimen | 1.49 | 2.02 | 0.295 | 0.54-7.50 |
|  | Sex (Female) | 0.97 | 1.01 | 0.902 | 0.36-3.15 |
|  | Year of treatment | 0.97 | 0.86 | 0.459 | 0.58-1.27 |
|  | Year of birth | 0.99 | 0.99 | 0.804 | 0.93-1.05 |
| Unknown | Short regimen | 0.43 | 0.68 | 0.734 | 0.08-5.87 |
|  | Sex (Female) | 0.91 | 0.95 | 0.956 | 0.17-5.19 |
|  | Year of treatment | 0.73 | 0.80 | 0.487 | 0.42-1.50 |
|  | Year of birth | 0.98 | 0.98 | 0.750 | 0.89-1.08 |

n=189; RRR: relative risk ratio  aRRR: adjusted relative risk ratio

**Sensitivity analyses**

**Table S3**. Poisson regression to evaluate the effect of the regimen of treatment, year of birth, sex, and year of treatment on the frequency of treatment completion. This model includes the unknow category as treatment completed.

| **Treatment complete** |  | **IRR** | **aIRR** | **p value** | **95% CI** |
| --- | --- | --- | --- | --- | --- |
| No (Ref outcome) |  | 1 | 1 | - | - |
| Yes | Short regimen | 1.03 | 1.05 | 0.786 | 0.72-1.55 |
|  | Sex (Female) | 0.99 | 1.00 | 0.978 | 0.74-1.36 |
|  | Year of treatment | 0.99 | 0.99 | 0.828 | 0.88-1.11 |
|  | Cohort | 0.99 | 0.99 | 0.973 | 0.85-1.17 |

n=189; RRR: relative risk ratio

**Table S4**. Poisson regression to evaluate the effect of the regimen of treatment, year of birth, sex, and year of treatment on the frequency of treatment completion. This model includes the unknow category as not treatment completed.

| **Treatment complete** |  | **IRR** | **aIRR** | **p value** | **95% CI** |
| --- | --- | --- | --- | --- | --- |
| No (Ref outcome) |  | 1 | 1 | - | - |
| Yes | Short regimen | 1.03 | 1.11 | 0.586 | 0.75-1.66 |
|  | Sex (Female) | 0.99 | 1.01 | 0.949 | 0.74-1.38 |
|  | Year of treatment | 0.99 | 0.99 | 0.886 | 0.88-1.12 |
|  | Cohort | 0.99 | 1.00 | 0.996 | 0.85-1.18 |

n=189; RRR: relative risk ratio
